# Stem cell specification and niche formation in developing incisor require actomyosin forces

**DOI:** 10.1093/stmcls/sxaf074

**Published:** 2025-11-26

**Authors:** Yasmin Mohtadi Hamadani, Laura Evers, Satu-Marja Myllymäki, Emma Juuri, Maria Jussila, Paul Gueguen, Mina Mina, Irma Thesleff, Anamaria Balic

**Affiliations:** Institute of Oral Biology, Centre for Dental Medicine, University of Zurich, Zurich 8032, Switzerland; Institute of Oral Biology, Centre for Dental Medicine, University of Zurich, Zurich 8032, Switzerland; Stem Cells and Metabolism Research Program, Faculty of Medicine, University of Helsinki, Helsinki 00014, Finland; Cleft Palate and Craniofacial Center, Department of Plastic Surgery, Helsinki University Central Hospital, Helsinki 00029, Finland; Orthodontics, Department of Oral and Maxillofacial Diseases, University of Helsinki, Helsinki 00014, Finland; Institute of Biotechnology, HiLIFE, University of Helsinki, Helsinki 00790, Finland; Functional Genomics Center Zurich, ETH Zurich and University of Zurich, Zurich 8057, Switzerland; Department of Pediatric Dentistry, University of Connecticut Health Center, Farmington, CT 06030, United States; Institute of Biotechnology, HiLIFE, University of Helsinki, Helsinki 00790, Finland; Institute of Oral Biology, Centre for Dental Medicine, University of Zurich, Zurich 8032, Switzerland

**Keywords:** continuously growing incisor, stem cell niche formation, single-cell RNA sequencing, actomyosin force, Sox2

## Abstract

**Background:**

The precise timing of stem cell specification and niche formation during murine incisor development is poorly understood, and it is unclear whether these processes occur simultaneously or in a sequential manner. Functional dental epithelial stem cells are marked by the expression of Sox2, a transcription factor that is broadly expressed in the dental epithelium at the dentition onset and restricted to stem cells in fully developed incisor.

**Methods:**

Using genetic lineage tracing in Sox2^*CreERT2/+*^; R26R^*mT/mG*^ and Sox2^*CreERT2/+*^; R26R^*tdT/+*^ embryos along with a single-cell RNA sequencing at different stages of incisor development, we investigated the timing of the stem cell specification and its temporal relationship with niche formation.

**Results:**

Our results reveal the presence of a Sox2-expressing stem cell-like population prior to formation of the functional niche. These cells localize to the leading edge of the advancing incisor epithelium where they are maintained in an undifferentiated state. Our data demonstrate presence of actomyosin network and a generation of a contractile tension, which helps confine Sox2^+^ stem cells to the leading edge.

**Conclusion:**

This mechanical confinement likely plays an important role in maintaining their stemness until the niche is functionally and structurally established. Partial or complete disruption of the actomyosin network disables the clustering of Sox2-expressing cells, potentially triggering their premature differentiation, and ultimately leads to impaired formation of the functional stem cell niche and abnormal growth of the incisor.

## Graphical abstract

**Graphical Abstract:**
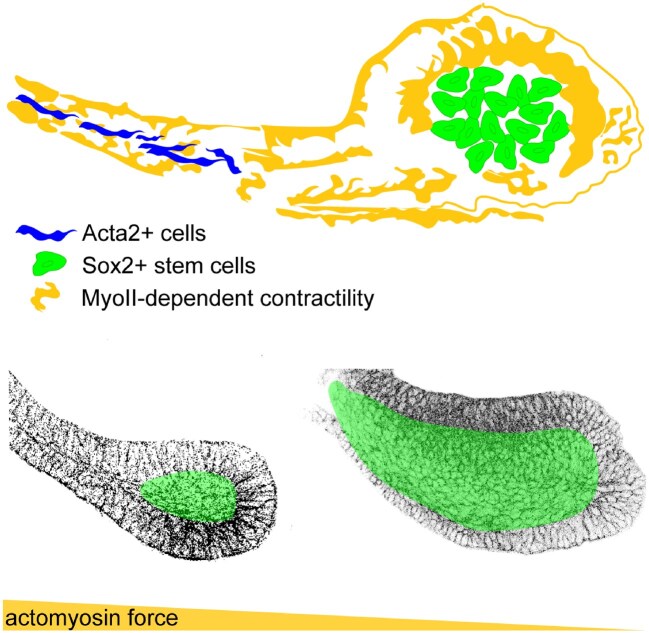

Significance statement Majority of organs develop systems that enable repair or regeneration of cells lost due to damage. These systems heavily rely on tissue-specific stem cells, but how and when these cells arise during organ development is not known. Here we use continuously growing mouse incisor, a paradigm of a regrowing organ, to dwell into stem cell specification and formation of the functional stem cell niche. We show that stem cells are specified prior to niche formation and are maintained in an undifferentiated state by biomechanical cues. These are intriguing findings that indicate a communication of molecular and mechanical cues that can be utilized toward generating functional organs *in vitro*.

## Introduction

The function and regeneration of many adult organs depend on resident stem cells, located within discrete anatomical spaces called niches; dynamic structures composed of cells and extracellular matrix that control the stem cell activity and behavior.^1^ Over the years, various epithelial stem cell niches have been discovered in several epithelial tissues, including the hair bulge in the skin, the intestinal crypt in the intestine and the cervical loop in the rodent incisor tooth.^2^^,^^3^ These niches share a similar structure in which epithelial stem cells are surrounded by their progeny and mesenchymal cells which collectively regulate their survival, proliferation, and differentiation.^1^^,^^4^ While extensive studies elucidated the distinct microenvironment that constitutes the stem cell niche, the timing and the regulatory machinery governing their formation remain elusive.

**Figure 1.**
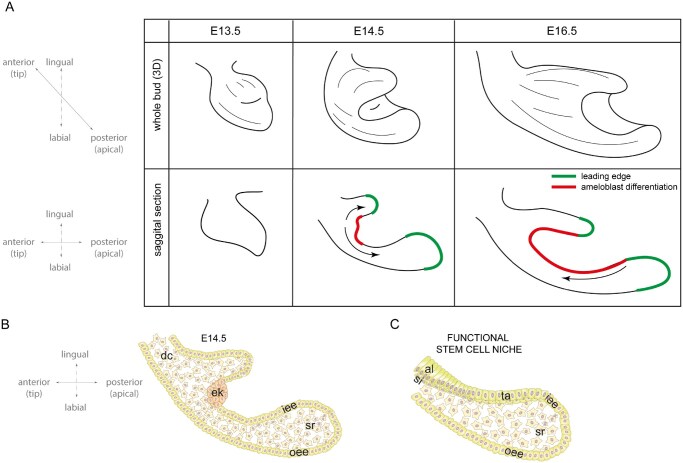
Illustration of incisor development. (A) Depiction of incisor development in 3D (upper row) and in cross section (lower row). The orientation is indicated on the left. The leading edges are shown in green and the inner enamel epithelium undergoing differentiation is shown in red. The arrow points to the direction of the differentiation. Cellular compartments in the incisor at E14.5 (B) and in the cervical loop at E16.5 (C). Abbreviations: iee = inner enamel epithelium, oee = outer enamel eptihelium, sr = stellate reticulum, al = ameloblast differentiation, si = stratum intermedium, ta = transit-amplifying cells, ek = enamel knot. Schematic in 1C is adapted from Binder et al.^17^

Mouse incisors are unique in that they continuously grow, which is supported by life-long preservation of epithelial and mesenchymal stem cells in these teeth. Unlike the mesenchymal stem cells, which are mainly recruited into the tooth,^5–8^ the incisor epithelial stem cells reside at the apical end of the tooth, in the *Notch1-*expressing stellate reticulum compartment of the cervical loop.^2^^,^^9^^,^^10^ Cervical loops first appear around embryonic day (E) 14.5, during the cap stage of the incisor development and constitute the leading edges of the tooth epithelium which extends in the apical direction (Figure 1A and B). These newly established cervical loops are composed of inner and outer enamel epithelium that enclose loosely arranged stellate reticulum cells^2^ (Figure 1B). While it is postulated that the appearance of cervical loops signifies established stem cell niches,^11^ there is no functional evidence to support this. Furthermore, ameloblast differentiation commences at the tip of the incisor at the early bell stage and advances toward the cervical loops,^2^ which suggests that the newly established cervical loops are not yet functional (arrows in the middle panel in Figure 1A). By E16, the cervical loops acquire their typical cellular architecture (Figure 1C) and exhibit gene expression profile characteristic of the functional stem cell niche. Specifically, *Sox2* expression becomes restricted to the stellate reticulum in the tip of the cervical loop, overlapping with other stem cell markers that also appear in the loop around this stage, including Lgr5, Bmi1, and Oct4.^12–14^ At the same stage, *Sfrp5* expression is detected in the inner enamel epithelium adjacent to the *Sox2* expressing domain,^12^ and *Shh* and YAP expression domains contiguous to it mark the presence of transit-amplifying cells.^13^^,^^15^ This suggests initiation of Sox2 lineage progression from the cervical loops and the shift of the direction of ameloblast differentiation from a posterior to an anterior trajectory, toward the tip (arrows in right side panel in Figure 1A). Additionally, *Fgf10* expression detected in the adjacent mesenchyme^16^ suggests a completion of the niche formation and the establishment of a functional microenvironment that supports and maintains epithelial stem cells. However, the functional data to support this hypothesis are missing.

Epithelial stem cells are marked by expression of *Sox2*, a transcription factor that is continually expressed in the developing incisor.^12^ Initially, it marks most of the cells in the primary dental lamina, after which the expression dynamically and gradually changes and becomes more restricted.^11^^,^^12^ It is found in the newly formed cervical loops at E14.5, and it persists in the cervical loops throughout life. This intriguing expression pattern of *Sox2* in the developing incisors raises the question of when *Sox2* expression signifies a stem cell phenotype in the dental epithelium. Tissue-specific stem cells do not exist prior to organ formation and are specified during organogenesis.^18^ Several studies have shown that Sox2 is essential for both the incisor formation and maintenance of epithelial stem cells and their niche.^11^^,^^19^ Conditional deletion of *Sox2* from the *Pitx2* expressing dental epithelium results in the incisor development arrested at E16.5, followed by gradual regression and disappearance of incisors by postnatal day (P) 0.^19^ Similarly, conditional deletion of *Sox2* using the *Shh^Cre^* mouse model impairs incisor growth and size, accompanied by progressive dysmorphogenesis after E15.5.^11^

In this study, we investigate the functional and molecular properties of the Sox2-expressing cells in the developing incisors using lineage tracing analyses and single-cell RNA sequencing. Our results demonstrate the presence of a stem cell-like population prior to niche formation. This population is found in the newly formed cervical loops at E14.5 and is maintained at the leading edge of the extending epithelium by a contractility force involving Myosin II (MyoII). The tension forces generated by this network confine Sox2-expressing stem cells at the leading edge of the advancing epithelium and hinder their differentiation until the establishment of the stem cell niche around E16. Partial or complete disruption of the actomyosin network *in vivo* and *in vitro* leads to premature differentiation of Sox2-expressing stem cells and impairs formation of the functional incisor stem cell niche and its hallmark cellular architecture.

## Results

### Sox2-expressing stem cells are established prior to completion of the niche formation

During incisor development, the *Sox2* expression domain dynamically changes its location and boundaries, initially labeling the majority of dental lamina and placode cells^11^^,^^12^^,^^20^ after which it gradually restricts to the labial edge of the advancing epithelium (Figure S1A–C, see online supplementary material for a color version of this figure). By E16.5, Sox2 expression is found exclusively in cervical loops which at this stage assume the morphology of the functional niche and are composed of all four tissue layers, including inner enamel epithelium, stratum intermedium, stellate reticulum, and outer enamel epithelium^12^ (Figure 1C and Figure S1D, see online supplementary material for a color version of this figure). This distinct spatiotemporal expression pattern of *Sox2* raises the question of the precise developmental stage at which Sox2-expressing cells acquire stem cell identity in the developing incisor. To address this, we analyzed the Sox2 lineage at different stages of incisor development using Sox2^*CreERT2/+*^; R26R^*mT/mG*^ and Sox2^*CreERT2/+*^; R26R^*tdT/+*^ embryos. A single tamoxifen injection was used to induce sparse labeling, which allows more accurate monitoring of individual clones and their expansion.^21^

**Figure 2.**
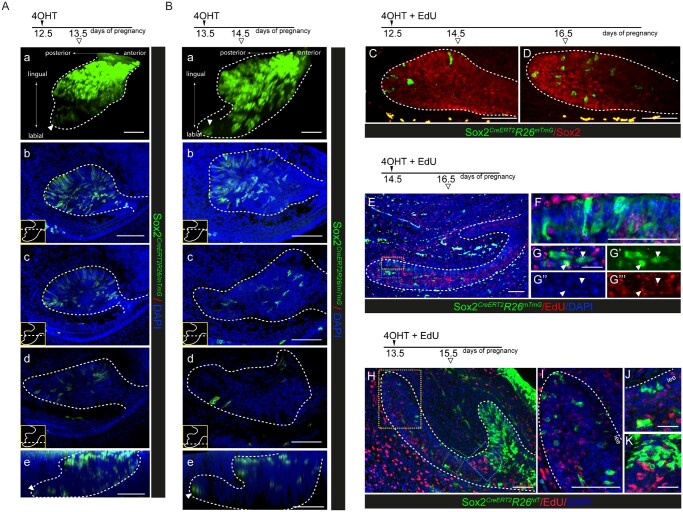
Lineage tracing of Sox2-expressing cells during incisor development. (A, B) 3D rendering (a) and optical sections in planar (b–d) and sagittal (e) views of confocal immunofluorescent images of whole mount samples of Sox2^*CreERT2/+*^; R26R^*mT/mG*^ incisors obtained from E13.5 (A) and E14.5 (B) embryos exposed to tamoxifen 24 h earlier. Schematics on the top outline the experimental setup. The boxes in the lower left corner in b, c, and d indicate the level of the optical section in the bud. Green color represents Sox2-labeled progeny, while nuclei are identified by DAPI. (C, D) Sox2 fluorescent immunostaining in paraffin sections of the leading edge from E14.5 (C) and E16.5 (D) Sox2^*CreERT2/+*^; R26R^*mT/mG*^ incisors exposed to tamoxifen at E12.5. Green color represents Sox2-labeled progeny, while red marks Sox2+ cells, and nuclei are identified by DAPI. (E-K) Immunofluorescence in tissue section of paraffin embedded E16.5 (E-G’’’) and E15.5 (H-K) incisor obtained from Sox2^*CreERT2/+*^; R26R^*mT/mG*^ (E-G’’’) and Sox2^*CreERT2/+*^; R26R^*tdT/+*^ (H-K) embryos exposed to tamoxifen and EdU at E14.5 (E-G’’’) or E13.5 (H-K). The schematic above E and H outlines the experimental setup. F and G-G’’’ are magnified views of the yellow and blue boxed areas in E, respectively. I, J, and K are magnified views of the yellow, green, and blue boxed areas in H, respectively. Green color represents Sox2-labeled progeny, red represents EdU+ cells, and DAPI identifies the nuclei. Dashed lines outline the incisor epithelium. Scale bar represents 100 µm.

We first traced Sox2 lineage between E12.5 and E13.5, during which time *Sox2* expression domain encompasses a large part of the tooth germ (Figure S1A, see online supplementary material for a color version of this figure). It is expressed by cells located in the posterior portion of the tooth germ that actively participate in the formation of the bud, that is completed by E13.5.^20^ Indeed, lineage tracing of Sox2-expressing cells in Sox2^*CreERT2/+*^; R26R^*mT/mG*^ embryos from E12.5, showed that a greater portion of the bud at E13.5 is composed of GFP+ cells (Figure 2A). The majority of GFP+ cells was localized on the lingual side of the bud (Figure 2A, a-e), and only a small portion was found on the labial side of the bud, localized in the leading edge of the epithelium (arrowhead in Figure 2Aa and e). Immediately following bud formation at the onset of cap stage, the *Sox2* domain splits between the two locations, one on the lingual side of the germ, and the other in the tip of the advancing labial epithelium (Figure S1B, see online supplementary material for a color version of this figure). These two domains are not completely separated and are interconnected at the buccal side of the tooth. We labeled Sox2-expressing cells at E13.5 and traced them over 24 h, which showed that the distribution of GFP+ cells did not significantly change (Figure 2B). The majority of GFP+ cells was located in the lingual aspect of the tooth germ, and only a small number was scattered across the labial side (Figure 2B, a-e). Additionally, there was an isolated subset of GFP+ cells at the newly formed cervical loops at the leading edge of the labial side (arrowhead in Figure 2Ba and e) which at E14.5 is composed mainly of *Sox2*-expressing cells (Figure S1E and F, see online supplementary material for a color version of this figure).

Majority of Sox2 progeny traced from E12.5 to E14.5 and located in the leading edge of the labial side was marked by Sox2 expression (Figure 2C). Furthermore, the labeled progeny maintained their Sox2 expression until E16.5 (Figure 2D). We therefore traced Sox2 lineage from E14.5 and analyzed the proliferation status of labeled cells by concomitant injections of EdU and 4-hydroxytamoxifen (4-OHT) to pregnant females at E14.5. At E16.5, the labeled Sox2 progeny was found mainly in the posterior portion of the labial side epithelium, including the cervical loop where they were found in all cell compartments (Figure 2E). In addition, clusters of labeled cells reminiscent of clonal expansion were found in the inner enamel epithelium in the cervical loop (yellow dotted box in Figure 2E and F). While most of the GFP+ cells in the cervical loop did not retain EdU, there were some GFP+ cells located within the stellate reticulum and outer enamel epithelium that did (blue dotted box in Figure 2E and G-G’’’). The EdU label retention and the scattered distribution of GFP+ cells suggest that the stem cell-like Sox2 progeny from E14.5 maintains quiescent state. This was further corroborated by 48 h Sox2 lineage tracing initiated at earlier time-point, at E13.5, which showed that labeled Sox2 progeny on the labial side of the incisor was mainly EdU negative (Figure 2H and I). In these samples, individual labeled cells were also found in the inner enamel epithelium (Figure 2I), and the rare clusters were located very close to the enamel knot (green dotted box in Figure 2H and J). Interestingly, a clear delineation was observed between the lingual side, that was almost entirely composed of labeled Sox2 progeny, and the lingual side that contained significantly less labeled cells (Figure 1H). This delineation was also visible in the enamel knot (Figure 2H and K). Taken together, these data suggest that Sox2+ progeny on the labial side proliferates between E13.5 and E14.5, when it assumes a quiescent status reminiscent of stem cell-like features. EdU label retention indicates that these cells do not proliferate but advance apically toward the location of the future stem cell niche along with the extending epithelium. This is also supported by a distribution of labeled cells at E16.5 after 2 days of tracing, which is predominantly in the posterior portion of the incisor epithelium, closer to and within cervical loop. Additionally, the presence of clusters of labeled cells in the inner dental epithelium within the cervical loop at E16.5, and absence of similar pattern at E15.5 indicates that functional stem cell niche is formed between E15.5 and E16.5.

### Comparative analyses of Sox2+ dental epithelial stem cells during incisor tooth development

**Figure 3.**
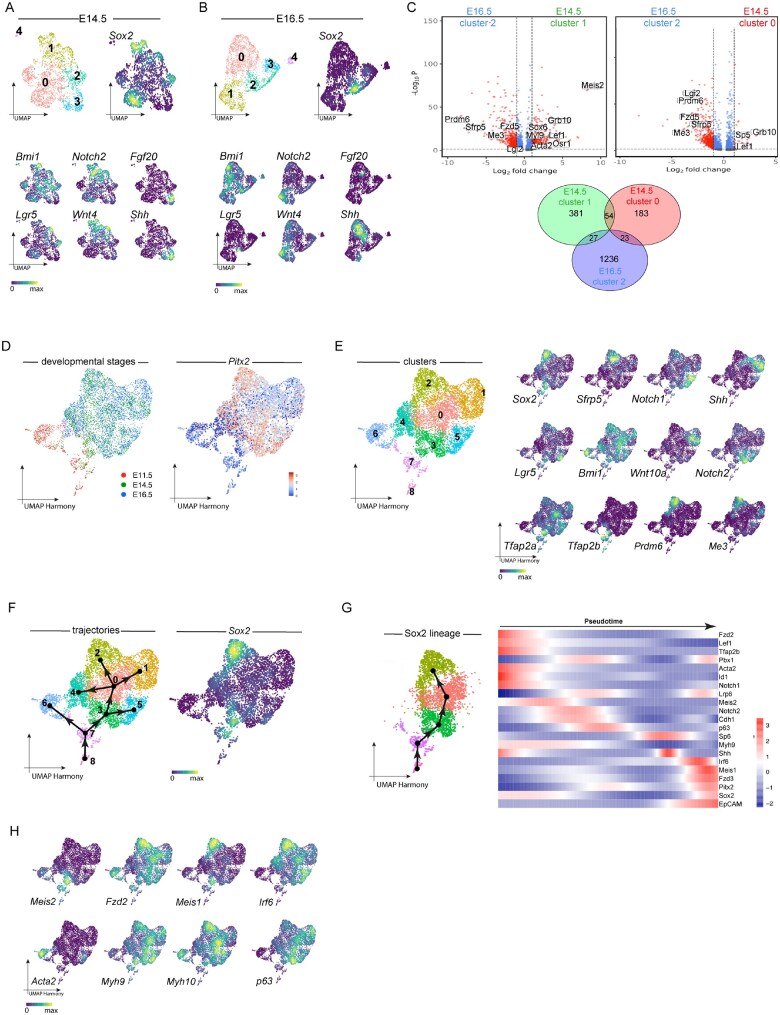
Single-cell RNA sequencing and transcriptome analyses of incisor epithelium at different developmental stages. (A, B) Visualization of cell clusters in UMAP plots and distribution of the expression of selected markers in UMAP-plotted clusters in the incisor epithelium at E14.5 (A) and E16.5 (B). (C) Volcano plot depicting comparison of differentially expressed genes in *Sox2+* cells from cluster 2 at E16.5 (left side in each panel), and clusters 1 (right side in left panel) and 0 (left side in right panel) at E14.5. Differentially expressed genes were computed using Wilcoxon rank-sum test. Volcano plots display genes with significance cutoffs of *P*-value < .05 and fold change > 1.0. Venn diagram below the panels shows the distribution of differentially expressed genes between these clusters. (D, E) Integrated analysis of incisor epithelium from three stages (E11.5, E14.5, and E16.5). (D) UMAP plot depicting the distribution of developmental stages (left) and *Pitx2* expression (right) in the integrated sample. (E) UMAP plots to visualize cell clusters (left) and distribution of the expression of selected markers (right) in the integrated sample. (F-H) Trajectory analysis and computational modeling of *Sox2* lineage. (F) UMAP plots showing the trajectories (left) and distribution of *Sox2* (right) in the integrated sample. (G) UMAP plot (left) and heatmap (right) presentation of *Sox2* lineage trajectory and differentially expressed genes. (H) UMAP plot visualization of expression of selected markers specifically expressed in the computational *Sox2* lineage trajectory.

The presence of quiescent stem cell-like population among the Sox2 progeny at E14.5 suggests potential molecular similarities with the Sox2+ stem cells at E16.5. We therefore performed single-cell RNA sequencing (scRNAseq) of incisor tooth germs from E14.5 and E16.5, as well as whole mandibles from initiation stage (E11.5) (Figure S2 [see online supplementary material for a color version of this figure] the numbers of cells analyzed are indicated in Figure S2M, see online supplementary material for a color version of this figure). In each sample we identified dental epithelium cluster by exclusive expression of *Pitx2*, in addition to *K14*, *p63*, *Sox2*, *EpCAM*, and other genes reportedly expressed in the dental epithelium (Figure S2, see online supplementary material for a color version of this figure).^2^ From here onward, our analyses focused exclusively on dental epithelium clusters (the numbers of cells are indicated in Figure S2M, see online supplementary material for a color version of this figure) from each stage and the *Sox2+* population within it (Supplemental Data S1-S3). At the E11.5, *Sox2+* cells were located in one of the two clusters within the *Pitx2+* dental epithelium, that was marked by expression of various Wnt (*Wnt3*, *Wnt4*, *Wnt7b*, etc) and Fgf (*Fgf8*, *Fgf9*, *Fgfr2*, etc.) signaling related molecules, *Shh*, and others (Figure S2A [see online supplementary material for a color version of this figure] and Supplemental Data S1). At E14.5, high *Sox2* expression was found in two distinct clusters, clusters 0 and 1, while low levels were found in cluster 2 (Figure 3A and Supplemental Data S4), which reflects the distribution of *Sox2* gene expression in the developing incisor at this stage (Figure S1C, see online supplementary material for a color version of this figure). Cluster 0 was marked by low expression of *Lgr5* and *Bmi1*, which were also found in cluster 1 (Figure 3A and Supplemental Data S4). However, compared to cluster 0, cluster 1 was enriched for markers of cell adhesion, migration, proliferation and epithelial differentiation (Figure S3A, see online supplementary material for a color version of this figure), as well as the exclusive *Wnt4* and *Notch2* expression, which indicates a suprabasal/stellate reticulum cell compartment (Figure 3A and Supplemental Data S4). Cluster 2 resembled cluster 1 in that it was enriched for markers of cell adhesion, migration, proliferation and epithelial differentiation (Figure S3B, see online supplementary material for a color version of this figure). At E16.5 most of *Sox2*+ cells localized in cluster 2 (Figure 3B and Supplemental Data S5), reflecting *Sox2 in vivo* expression domain found exclusively in the established cervical loops (Figure S1D, see online supplementary material for a color version of this figure).

Comparative gene expression analyses of *Sox2+* cells from different stages indicated highest transcriptome similarities between E14.5 and E16.5 (40.5%), while only 17.7% of genes were shared between E14.5 and E11.5 (Figure S3C-F, see online supplementary material for a color version of this figure). Furthermore, comparative analysis of individual *Sox2+* clusters from E14.5 and *Sox2+* cells from E16.5 (cluster2) showed that each cluster from E14.5 shared similar number of genes with the cluster 2 from E16.5 (Figure 3C). However, they differ in the number of exclusively expressed genes, that was lower in cluster 0 (183 genes) compared to cluster 1 (381 genes) (Figure 3C, lower panel). Additionally, the cluster 2 from E16.5 was marked by significantly upregulated expression of *Sfrp5* (Figure 3C), a marker of early progeny of *Sox2+* stem cells.^12^ Presence of this marker indicates activated differentiation of stem cells and functional stem cell niche. Taken together, these analyses suggest that *Sox2+* cells from E14.5 are stem cells which preserve their molecular profile until the formation of the niche by E16.5. This further suggest that formation of cervical loops at E14.5 does not correlate with the formation of the functional stem cell niche.

Integrative analysis of the dental epithelium clusters from all stages (Figure 3D) demonstrated distribution of *Sox2* expression between the clusters 2, 3, and 7 (Figure 3E). Clusters 3 and 7 were composed mainly of cells from E11.5, as well as fewer cells from E14.5 (Figure 3D), and were marked by the expression of *Lgr5*, *Bmi1*, *Tfap2a*, and *Tfap2b* (Figure 3E). Cluster 2 was composed mainly of *Sox2*+ cells from E14.5 and E16.5 incisors (Figure 3D) and was further characterized by the expression of stem cell markers *Lgr5* and *Bmi1*, as well as *Prdm6* that was found significantly upregulated in *Sox2+* cells at E16.5 (Figure 3E and C). Immediately adjacent to cluster2 were the cells expressing *Sfrp5*, *Notch1*, and *Shh.* This suggested that clusters 3 and 7 are the start of the *Sox2* lineage that ends in cluster 2 which represents established stem cell niche and was confirmed by *Sox2* lineage differentiation trajectory (Figure 3G). Taken together, these data corroborate our lineage tracing experiments and further support the hypothesis that incisor epithelial stem cells are established prior to stem cell niche formation, at E14.5.

### Actomyosin network in the dental epithelium confines Sox2-expressing cells to the leading edge of the labial epithelium

**Figure 4.**
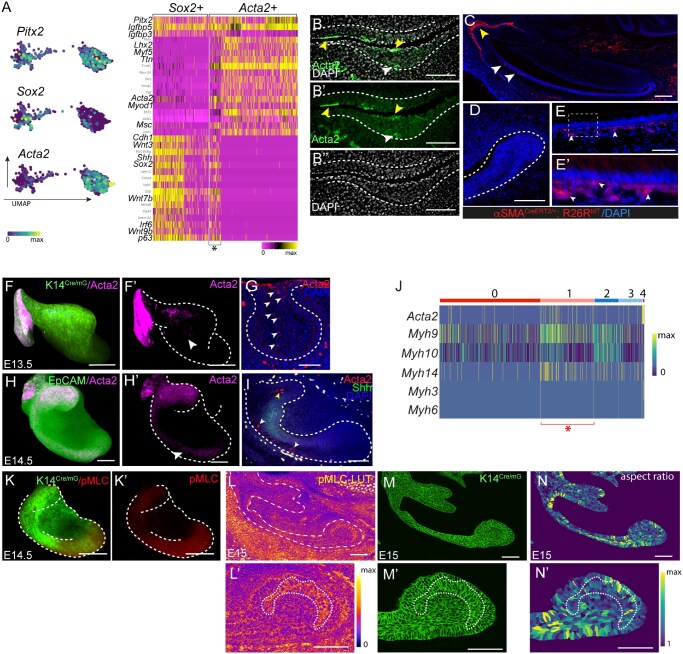
Contractile force in the incisor development. (A) Distribution of the expression of selected markers in UMAP-plotted clusters and heatmap distribution of genes (lower panel) in the dental epithelium at E11.5. (B-B’’) Immunofluorescence in tissue section of paraffin embedded mandibles at E11.5 showing the expression of Acta2 in the thickened dental epithelium. Nuclei are stained by DAPI. (C-E’) detection of Acta2 progeny in E17.5 incisors obtained from αSMA^*CreERT2/+*^; R26R^*tdT/+*^ embryos exposed to tamoxifen at E11.5. (F-I) Analysis of Acta2 expression in the developing incisor. Confocal images of whole mount fluorescent immunostaining (F, F’, H, and H’) and immunofluorescence in paraffin sections (G, I) in developing incisors at E13.5 (F-G) and E14.5 (H-I). 3D surface rendering of incisor epithelium (F, F’, H, and H’) depicting Acta2 expression in the incisor epithelium detected by K14^Cre/mG^ (F) or EpCAM (H) staining. Immunofluorescence staining in paraffin sections of E13.5 (G) and E14.5 (I) incisor against Acta2 (G, I) and Shh (I). Nuclei are stained by DAPI. (J) Heatmap depicting expression of selected genes per cell of the E14.5 dental epithelium from Figure 3A. (K-M’) Analysis of pMLC expression in the developing incisor. Confocal images of fluorescent immunostaining in whole mounts (K, K’) and frozen sections (L-N’) of E14.5 (K, K’) and E15 (L-N’) incisors. 3D surface rendering of incisor epithelium (K, K’) identified by K14^Cre/mG^ (K) and depicting pMLC expression . pMLC (L, L’), K14^Cre/mG^ (M, M’), and aspect ratio analysis (N, N’) in frozen section. L’, M', and N’ are high magnification images of apical edge from L, M, and N. Scale bar represents 100 µm.

Interestingly, scRNAseq analysis showed that at E11.5, *Pitx2+* dental epithelium grouped in two clusters, one which predominantly contained *Sox2*, *Shh*, and *p63-*expressing cells, and the other marked by contractility related genes, such as *Acta2*, *Myl9*, *Myod1*, *Myf5*, and others (Figure 4A and Supplementary Data). A small subset of cells within this cluster showed an expression profile similar to that of the *Sox2+* cluster (asterisk in Figure 4A). *Acta2* labels epithelial stem cells in the adult incisors,^22^ which is why we further analyzed its expression and contribution to stem cells during incisor development. At E11.5 Acta2+ cells were found in the periderm, as previously reported,^23^ but also in the basal layer of the epithelial thickening (Figure 4B-B’’). Lineage tracing of Acta2+ cells from E11.5, using Acta2-*Cre* mouse line (from here onward referred to as αSMA^CreERT2^ line) showed no tdT+ cells in the cervical loops of αSMA^CreERT2^; R26^tdT/+^ incisors at E17.5 (Figure 4C and D). Instead, tdT+ cells contributed to distinct epithelial compartments, including epithelial cap, dental cord (yellow arrowhead in Figure 4C), and the outer enamel epithelium and stratum intermedium of the distal end of the incisor (white arrowheads in Figure 4C and E-E’). Interestingly, at E13.5 Acta2+ cells displayed columnar arrangement spanning the full height of the central bud core (Figure 4F and G) from which they extended into the leading edges of the advancing epithelium on both labial (white arrowheads in Figure 4H and I) and lingual (yellow arrowhead in Figure 4I) sides by E14.5. On either side, Acta2+ cells were found in the stellate reticulum located behind the Shh expression domain marking the enamel knot and the differentiating inner dental epithelium (Figure 4I). This was also confirmed by our scRNAseq data set from E14.5, which showed *Acta2+* cells present in high numbers in cluster 1 marked by stellate reticulum identity (Figure 4J). Interestingly, this cluster was also marked by high number of cells expressing non-muscle myosin related genes, such as *Myh9*, *Myh10*, and *Myh14* (Figure 4J).

We therefore asked a question whether Acta2 expression reflects a requirement to generate contractile forces in the dental epithelium. To assess contractility, we stained for phosphorylated myosin light chain (pMLC) that activates MyoII motors, facilitating contraction of actin filaments.^24^ At E14.5, high levels of pMLC were distributed in a pattern that overlapped but was more extensive than Acta2 labeling (Figure 4K-K’), as expected due to the expression of additional actin isoforms that also engage MyoII motors. At E15, at which time the leading edge is actively moving posteriorly, pMLC was detected on the labial side of the incisor epithelium and in the adjacent mesenchyme (Figure 4L). At the leading edge, pMLC staining was observed as a continuous band, oriented perpendicular to the direction of epithelium elongation (Figure 4L’-N’).

**Figure 5.**
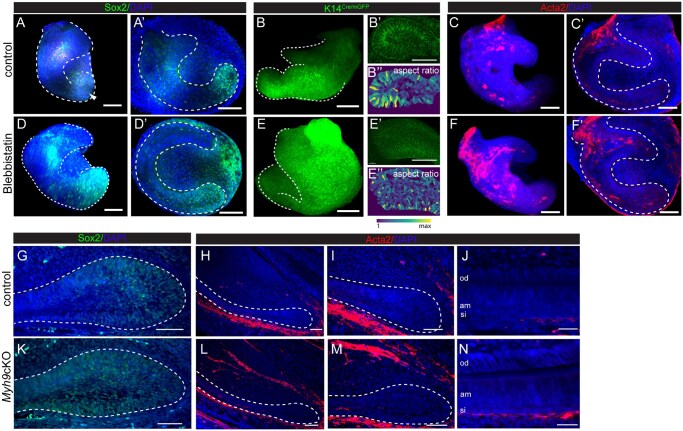
Effect of inhibition of non-muscle myosin II in developing incisor. (A-F’) Confocal images of fluorescent immunostaining in whole mount control (A, A’, B, B’, B’’, C, C’) and Blebbistatin (D, D’, E, E’, E’’, F, F’’)-treated explants. 3D rendering (A, B, C, D, E, F) and virtual optical sections (A’, B’, B’’, C’, D’, E’, E’’, F’) showing Sox2 (A, A’, D, D’), K14^Cre/mG^ (B, B’, E, E’), and Acta2 (C, C’, F, F’) expression. Cell shape analysis (B’’, E’’) in virtual sections from B’ and E’. (G-N) Immunofluorescence in paraffin sections from control (G-J) and *Myh9*cKO (K-N) incisors at E17.5 showing Sox2 (G, K) and Acta2 (H-J, L-N). Nuclei are identified by DAPI. Dashed lines outline the incisor epithelium. Scale bar represents 100 µm. Abbreviations: od = odontoblasts, am = ameloblasts, si = stratum intermedium.

To test whether contractility plays a functional role in the stem cell niche formation, we cultured incisor buds from E14.5 embryos in the presence of Blebbistatin, a small molecule inhibitor of MyoII,^25^ and analyzed the distribution of Sox2+ cells in these explants. Compared to control, in which Sox2+ cells were confined to the leading edge, in Blebbistatin-treated explants they extended across main portion of the labial side of the bud (Figure 5A, A’, D, D’). Furthermore, cells in the leading edge of Blebbistatin-treated samples displayed shape change, specifically a decrease in aspect ratio (Figure 5B-B’’, E-E’’). At the same time, number of Acta2+ cells in these explants was increased compared to control (Figure 5C, C’, F, F’).

Expansion of Sox2+ cells through the entire cervical loop region was also evident in E17 incisors obtained from embryos with conditional deletion of Myh9 in the dental epithelium (*Myh9*cKO) (Figure 5K). Myh9 and Myh10 encode non-muscle myosin heavy chains IIA and IIB,^26^ that based on our scRNA-seq dataset were the main MyoII isoforms expressed in the dental epithelium (Figure 3H). They facilitate the early invagination of the incisor epithelium,^27^ but their role in the stem cell specification and the formation of the stem cell niche is not known. Intriguingly, these incisors did not display scattered distribution of Acta2+ cells as seen in control incisors (Figure 5H-J). Instead, Acta2+ cells were organized in a strip adjacent to stratum intermedium, and running along the entire labial side, including the cervical loop (Figure 5L-N).

**Figure 6.**
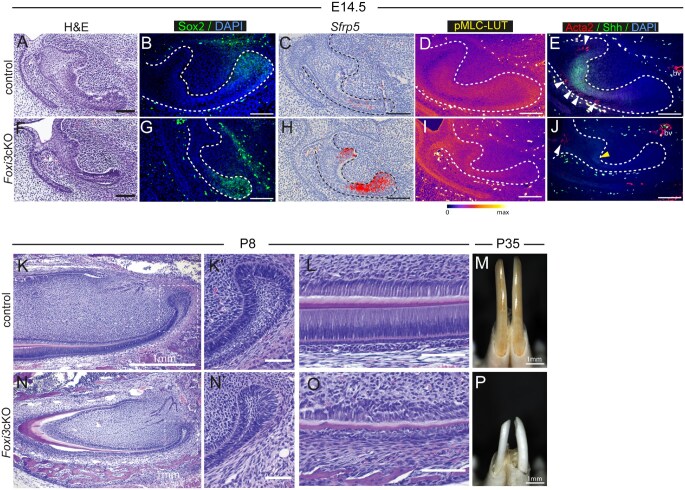
Sox2-expressing cells are not confined to stem cell niche in *Foxi3*cKO incisors. (A-P) Morphological (A, F, K-L, N-O) and molecular (B-E, G-J) analyses of *Foxi3*cKO incisors at E14.5 (A-J), P8 (K-L, N-O), and P35 (M, P). Hematoxylin and Eosin (H&E) staining (A, F, K-L, N-O), immunofluorescence (B, D, E, G, I, J), and radioactive *in situ* hybridization (C, H) in paraffin sections of control (A-E, K-L) and *Foxi3*cKO (F-J, N-O) incisors at E14.5 (A-J) and P8 (K-L and N-O) showing Sox2 (B, G), *Sfrp5* (C, H), pMLC (D, I), Acta2 (E, J), and Shh (E, J) expression. Nuclei are identified by DAPI. Dashed lines outline the incisor epithelium. Scale bar represents 100 µm. (M, P) images of 35-day-old incisors. Scale bar represents 1 mm.

Another mutant line we previously studied, similarly demonstrates an expanded Sox2 expression domain across the labial side. Incisors obtained from E14.5 mice in which Foxi3 is conditionally knocked out in the dental epithelium (*Foxi3*cKO) display changes reminiscent of those we previously published in the molar tooth buds,^28^ including depletion of suprabasal cells of the stellate reticulum and dental cord (Figure 6A and F). In contrast to controls at this stage, Sox2+ cells in *Foxi3*cKO incisors were spread across the labial side (Figure 6G), which coincided with precocious activation of *Sfrp5* that suggests a premature and ongoing differentiation of stem cells (Figure 6C and H). Interestingly, the pMLC levels in the leading edge of mutant teeth were significantly downregulated, while the levels in the mesenchyme were comparable to those observed in control incisors (Figure 6D and I). Intriguingly, we detected a very small number of Acta2+ cells in *Foxi3*cKO incisors, and only in the dental cord where pMLC levels were comparable to those of controls (Figure 6E and J). The Acta2 staining was not detected in the rest of the mutant tooth (Figure 6J). In addition, no significant reduction of pMLC was observed in the enamel knot area of mutant incisors (Figure 6I) despite the lack of Shh expression (Figure 6J). These changes lead to morphological and functional alterations of the stem cell niche and, ultimately, generation of smaller, misshapen, and grossly affected incisors (Figure 6K-P). Collectively, these data show that *Foxi3cKO* incisors lack the mechanism that confines Sox2+ stem cells to the leading edge of the labial side and maintains their stemness, which leads to their premature differentiation and impaired formation of a stem cell niche with proper structural and cellular organization.

## Discussion

The continuous growth of mouse incisors depends on the epithelial stem cells identified by the expression of Sox2 which is expressed in the tooth from the earliest stages of development. At the time of tooth initiation (E11.5), *Sox2* is expressed in the entire primary dental lamina, a strip of thickened epithelium representing the field of odontogenic activity that generates the entire dentition. By E16.5 *Sox2* expression is confined to the labial cervical loop where it is maintained in epithelial stem cells throughout life.^2^^,^^11^^,^^12^ This intriguing expression pattern raises a question of when during the incisor development Sox2 expression signifies the stem cell identity in the epithelium. We have addressed this question by performing lineage tracing and transcriptome analysis of Sox2-expressing cells at different stages of incisor development and show that Sox2 identifies a stem cell population as early as E14.5 that correlates with the timing of cervical loop formation. These stem cells remain in the newly formed cervical loops at the leading edge and are maintained as stem cells until the functional and structurally complete stem cell niche is established by E16.5. Moreover, we show that actomyosin forces are necessary to confine this population at the leading edge, and that lack of these forces leads to their premature differentiation and results in a formation of functionally and structurally impaired stem cell niche.

Transition from bud to cap stage during incisor tooth development is marked by the extension of epithelium away from the enamel knot and the formation of cervical loops at the leading edges.^2^ It has been postulated that the formation of cervical loops at E14.5 signifies the establishment of the stem cell niche,^11^ which was further supported by the presence of Sox2 expression in both labial and lingual cervical loops at E14.5.^12^ Our lineage tracing analyses showed that these cells do not participate in the ameloblast differentiation which commences at the tip of the incisor, based on lack of clonal expansion of labeled cells in the inner enamel epithelium of the cervical loop prior to E16.5. Clusters of Sox2 progeny in the inner enamel epithelium of the cervical loop were present in the traced samples at E16.5, which coincided with the detection of *Sfrp5+* early progeny in our scRNAseq set. This indicates that the functional stem cell niche forms at E16.5, which is further supported by reported appearance of Lgr5 and other stem cell markers^14^ and the rise of TA cells expressing Shh and YAP^13^^,^^15^ in the cervical loops at this stage. Additionally, our data show that the niche formation is preceded by specification of the Sox2+ stem cells responsible for incisor homeostasis and continuous growth. A small population of Sox2+ cells was maintained in the newly formed cervical loops, as evidenced by their low proliferation rate and EdU label retention by E16.5, during which time they maintained more than 40% of their transcriptional signature. This suggests that Sox2 expression in the newly formed cervical loops, at E14.5 marks the stem cell identity, but it does not exclude the possibility that stem cell identity already existed in the developing incisor. The early embryo does not contain tissue-specific stem cells prior to organ formation, which suggests that tissue-specific stem cells rise from the organ primordium either as a remnant or a differentiated progeny of the primordium cells.^18^ Some transcription factors, like Sox2 in the tooth, are essential for both the formation of a specific primordium and maintenance of the resulting stem cells.^12^^,^^18^^,^^29^ However, lower similarity in the transcription profile of Sox2+ cells from E11.5 and E14.5, compared to Sox2 populations from E14.5 and E16.5 does not support this possibility. Loss of Sox2 expression from the time of initiation displays profoundly detrimental effect on incisor development only after E16.5, while the effect on earlier stages is relatively mild, which further supports our conclusion.^11^^,^^19^

Our study shows that formation of stem cell niches and maintenance of Sox2+ stem cells in the newly formed cervical loops relies on contractile force. MyoII is critical for tooth bud invagination and morphogenesis,^27^ and earlier studies in zebrafish have suggested that actomyosin contractility conducted through formation of actin rings at the tissue margins enables extension of epithelial sheets.^30^ However, our study indicates that global MyoII inhibition in incisor explants *in vitro* did not significantly affect overall size or shape of the bud. Instead, these results demonstrated that contractile force is essential for the confinement of Sox2+ stem cells to the leading edge during epithelium extension posteriorly. Inability to confine Sox2+ stem cells to cervical loop was also observed in *Myh9*cKO incisors, albeit the effect was not as striking as *in vitro* inhibition of MyoII, most likely due to compensation from other MyoII constituents, like Myh10 and 14. Likewise, the extended distribution of Sox2+ cells on the labial side of incisor and their premature differentiation which we observed in *Foxi3*cKO incisors could be linked to decreased contractile force in the leading edge of this model.

The presence of contractility-related genes, such as *Acta2*, *Myh9*, *Myh10*, in our transcriptomics analyses at all stages of tooth development, including tooth initiation, points to a complex molecular network, that should be studied further. The most intriguing was Acta2 (also known as alpha-smooth muscle actin or α-SMA) expression, which has been associated mainly with stem cell populations in the tooth mesenchyme.^7^^,^^31^ More recently Acta2 was found in the epithelial stem cell niche of adult incisors, where it marked a very restricted stem cell population.^22^ In contrast, Ye et al. reported Acta2 expression specifically in the periderm dispersed over the oral and tooth placode epithelium.^23^ In our study we show both by immunostaining and scRNAseq that Acta2 expressing cells exist also in the dental epithelium, at all stages analyzed. Particularly intriguing is their columnar alignment in the core of the E13.5 incisor bud from which they extend toward cervical loops at the onset of their formation at E14.5. This arrangement suggests the presence of a columnar zone with distinct biomechanical properties, that potentially supports tissue architecture and mechanical signaling within the bud, thereby contributing to tooth morphogenesis. An elevated number of Acta2+ cells following MyoII inhibition in explants and in *Myh9*cKOs, suggests a potential regulatory feedback mechanism within the actomyosin network and highlights an intriguing possibility that Acta2 may exert compensatory functions, such as modulation of stiffness, stress fibers, and focal adhesions.^32^^,^^33^ It can therefore be speculated that strong actin accumulation observed in *Myh9/10^epi-cko^* mutants is in part related to augmented Acta2 expression.^27^ Future studies utilizing conditional or full Acta2 knockout will likely shed more light on its role in epithelial morphogenesis and biomechanical properties of tooth epithelium.

In conclusion, we propose a fundamental role for the actomyosin network in generating contractile force that maintain the spatial restriction of stem cells until a fully functional niche is formed. In this model, the leading edge advances in the apical direction, and the stem cells within it move along, pushed by the proliferating cells from behind. We also postulate that a transient population of Acta2+ cells plays a role in the developing incisor by supporting the mechanical constraints which confine stem cells to the leading edge. Loss of contractile force compromises the stability of the niche and depletes the tension forces which kept the stem cells restrained, ultimately leading to impaired development of the functional stem cell niche.

## Methods

### Animals

The animals used in this study include Sox2^*CreERT2/+*^,^34^ R26R^*mT/mG*^ (JAX stock 007576), R26R^*tdT*^ (JAX stock 007914), αSMA^CreERT2^ R26^tdT/+^,^31^ K14-Cre; Myh9^*fl/fl*^,^35^ K14-Cre; R26R^*mT/mG*^ and *K14-Cre43; Foxi3^fl/fl^*.^28^ Wild type NMRI mice were used to obtain embryonic tissue for single cell RNA sequencing (scRNAseq) and organ culture. All experimental procedures in this study were performed following protocols approved by the Finnish national animal experimentation board, under the animal license ESAVI/26019/2020. For the lineage tracing experiments, pregnant females were injected with Tamoxifen (75 mg/kg of body weight) with or without the EdU (25 mg/kg of body weight) at the specific times, indicated for each experiment.

### Tissue isolation and processing

The day of the plug was considered embryonic day (E) 0.5. Embryos were isolated in cold PBS and staged based on the limb and craniofacial morphology. *Paraffin embedding:* Whole heads of embryos younger than E14.5 or lower jaws were fixed overnight in 4% paraformaldehyde, pH7.4 at +4°C and then gradually dehydrated to 100% Ethanol and paraffin embedded, as previously described.^28^ Tissue was sectioned in 5-7 µm thick sections and processed for Hematoxylin and Eosin (H&E) staining, immunostaining or *in situ* hybridization. *Cryosectioning:* whole heads or mandibles obtained from E14.5 embryos were fixed for 2 h at 37°C, incubated in 30% sucrose until sinking and then frozen embedded in OCT media.

### Immunostaining and imaging

Immunostaining was performed on tissue sections as previously described^36^ or on incisor buds isolated from the lower jaws, and fixed overnight in 4% paraformaldehyde, pH 7.4 at +4°C. After washing in PBS, the buds were permeabilized in 0.5% Triton in PBS, blocked in 10% donkey serum, 3% BSA, 0.5% Triton in PBS for 6-8 h at +4°C and incubated with primary antibody overnight at +4°C. The following day primary antibody was washed with PBS and tissues were incubated with second primary antibody, overnight at +4°C, followed by PBS washes and incubation with secondary antibodies and Hoechst (1:2000) or DAPI (1:5000) stain. After overnight incubation at +4°C, the samples were extensively washed, mounted using Vectashield mounting medium, and imaged. Samples older than E13.5 and tissue explants were additionally cleared with 88% Histodenz in PBS before mounting. Primary Antibodies used were anti-Sox2 (AB5603, 1:200), anti-Acta2 (ab5694, 1:100), anti-pMLC (ab2480, 1:100), anti-Shh (AF464, 1:100), anti-Keratin (905301, 1:200), anti-GFP (ab13970, 1:200) and anti-RFP (STJ140000, 1:200). Secondary antibodies used were Alexa Fluor conjugated anti-rabbit (A-21206 or A-31573), anti-goat (A-21447 or A-11055) and anti-chicken (703-545-155), all in dilution 1:500. Tissue sections were imaged under the DM6000B Leica microscope, a standard upright fluorescence microscope equipped with DFC420C (color) and DFC350FX (B&W) Leica cameras. Whole mounts were imaged under the Leica Stellaris 5 automated upright confocal laser scanning microscope equipped with HC PL APO CS2 20x/0.75 NA Air and HC PL APO CS2 63x/1.3 NA Glycerol objectives or Olympus IXplore SpinSR10 spinning disk confocal microscope, equipped with two sCMOS cameras, a Yokogawa CSU-W1 50 μm pinhole disk and a 20x/0.8 NA air objective. Images were processed and analyzed using Bitplane Imaris 10.2.0 or Fiji software.

### *In situ* hybridization

Radioactive *in situ* hybridization was carried out according to a standard protocol.^28^ To detect gene expression the following [35S]-UTP (Perkin Elmer)-labeled RNA probes were used: *Sox2*,^37^  *Sfrp5*,^38^  *Notch1* and *Notch2*. Processed sections were imaged under a Zeiss Axio Imager M2 microscope equipped with an Axio Cam HR camera (Zeiss) and generated images were processed in Photoshop.

### Organ culture

Incisor buds were isolated from wild type embryos at E14.5 and cultured in a hanging drop. Culture media was composed of DMEM: F12 supplemented with 10% fetal bovine serum (FBS), Glutamax, Pen-Strep and ascorbic acid. Blebbistatin was added in 15 µmol/ml concentration. After the culture period of 3-5 days the samples were collected, fixed and processed for whole mount immunostaining as described earlier. Samples were imaged using Zeiss Lumar stereo microscope equipped with Apolumar S 1.2× objective.

### Isolation of cells for single cell RNA sequencing

Wild type mandibles or lower incisor buds were dissected from E11.5 or E14.5 and E16.5 embryos, respectively. Samples were obtained from three (E11.5 and E14.5) or two (E16.5) individual litters. In total 8 mandibles from E11.5, and incisor buds from 4 (E14.5) and 2 (E16.5) mandibles were individually processed for cell isolation. Each sample was incubated in Collagenase P (2.5U/ml of PBS) for 20-30 min at 37 °C with gentle rocking, to obtain single cell suspension. Following the incubation, the remaining tissue pieces were broken by gentle pipetting through narrowed Pasteur pipette and then cold DMEM containing 10% FBS was added to the samples to inactivate the enzyme. The samples were spun at 300 g for 5 min and the pellet was washed in cold phosphate buffer saline (PBS) supplemented with 0.04% bovine serum albumin (BSA), spun and resuspended in 50 µl of 0.04% BSA in PBS to reach the optimal concentration of 800-1100 cells/µl. The samples were then processed using the Chromium single cell 3’ library & gel bead Kit v3 (10X Genomics), and generated libraries were sequenced on an Illumina Novaseq 6000 (Illumina).

### Single cell RNA sequencing

Raw sequencing data from E11.5, E14.5, and E16.5 mouse dental tissues were processed using Cell Ranger (v4.0.0, 10X Genomics). Sequencing reads were aligned to the mouse reference genome (mm10-2020-A, supplied by 10X Genomics). Single-cell RNA sequencing data was processed using the Seurat v5 pipeline.^39^ Raw gene expression matrices were filtered to remove low-quality cells using standard quality control metrics: cells with fewer than 700 genes and/or mitochondrial content greater than 20% were excluded. Gene expression values were normalized using SCTransform^40^ and batch effects were corrected using Harmony integration.^41^ Principal Component Analysis (PCA) was performed on the normalized and batch-corrected data using the top 2000 variable genes. The optimal number of principal components (*n* = 20) was determined based on elbow plots. Uniform Manifold Approximation and Projection (UMAP) was used for dimensionality reduction. Clustering was performed using Seurat’s implementation of the Leiden algorithm (resolution = 0.4) on a shared nearest neighbor (SNN) graph constructed from the harmony-corrected PCA space.

#### Gene expression analysis

Differential gene expression analysis was performed using the Wilcoxon rank-sum test with a minimum log-fold change threshold of 0.25 and a minimum percentage of expressing cells of 10%. Volcano plots were generated using EnhancedVolcano with significance thresholds of *P*-value < .05 and fold change > 1.0. For trajectory-based differential expression, we employed tradeSeq^42^ to identify genes showing significant associations with pseudotime and differences between lineages. Key marker genes were visualized using Nebulosa’s plot_density function to show expression patterns across the UMAP space. Feature plots and violin plots were generated using Seurat’s visualization functions. For heatmaps, gene expression values were scaled and visualized using pheatmap with row-wise clustering and custom color schemes.

#### Cell population distance analysis

Mean squared error (MSE) distances were calculated between cell populations to quantify transcriptional differences across developmental stages. Mean expression vectors were computed for each population and pairwise MSE distances were visualized using pheatmap.

#### Trajectory analysis

Developmental trajectories were inferred using the Slingshot algorithm^43^ implemented in the slingshot R package. The analysis was performed using the harmony-corrected UMAP embeddings as the dimensionality reduction space. Cluster 8 was specified as the starting point and cluster 2 as the endpoint based on developmental timing and marker gene expression. Multiple lineages were identified and colored individually in trajectory visualizations.

#### Software and statistical analysis

All analyses were performed in R (version 4.1.0) using the following key packages: Seurat (v5.0.0) for single-cell analysis; slingshot (v2.0.0) for trajectory inference; tradeSeq (v1.6.0) for trajectory visualization; ggplot2 (v3.3.5) for visualization pheatmap (v1.0.12) for heatmap generation.

## Supplementary Material

sxaf074_Supplementary_Data

## Data Availability

The data underlying this article are available in the article and in its online supplementary material, except the transcriptomics data that are accessible at the GEO repository (GSE299463).
