# Supplementary material for: Stem cell specification and niche formation in developing incisor require actomyosin forces
Source: Stem Cells. 2025 Nov 26;44(2):sxaf074. doi: 10.1093/stmcls/sxaf074 (PMC12855154; doi:10.1093/stmcls/sxaf074)
Supplement: sxaf074_Supplementary_Data [file sxaf074_supplementary_data.zip › Supplementary Information.docx]

**Supplementary information**

**Stem cell specification and niche formation in developing incisor requires actomyosin forces**

***Yasmin Mohtadi Hamadani^1^, *Laura Evers^1^, Satu-Marja Myllymaki^2^, Emma Juuri^3,4^, Maria Jussila^5^, Paul Gueguen^6^, Mina Mina^7^, Irma Thesleff^5^, Anamaria Balic^1^**

**
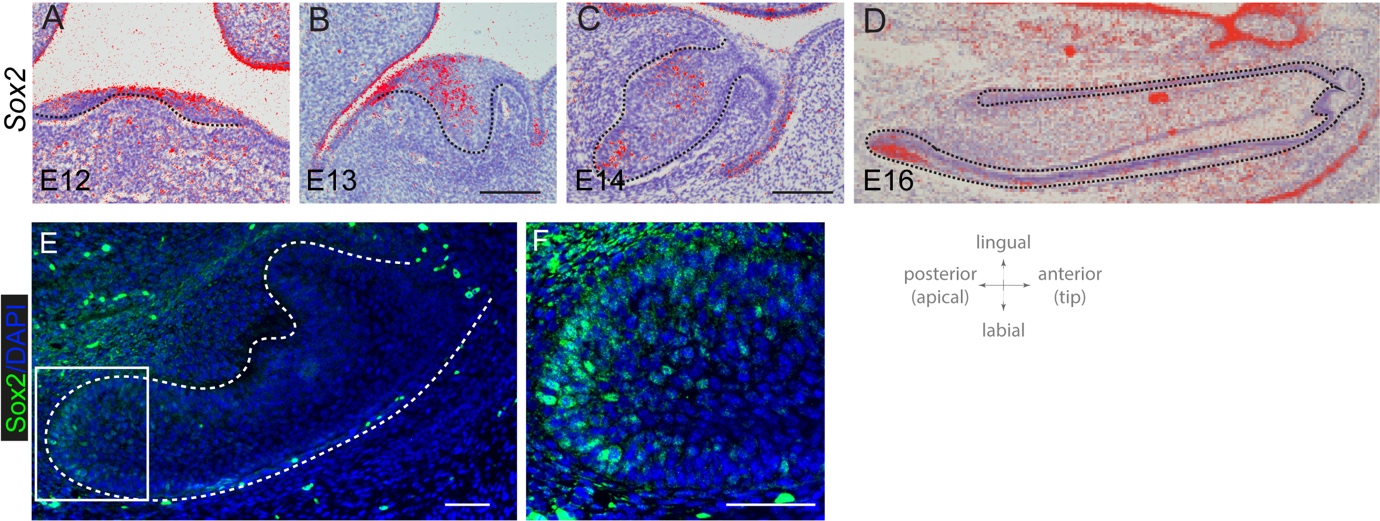
Supplemental Figure 1. Expression of *Sox2* during incisor development.** (A-D) Radioactive *in situ* hybridization of *Sox2* expression in the paraffin embedded tissue sections of developing incisors at E12, E13, E14 and E16. (E-F) Sox2 fluorescent immunostaining in paraffin section of E14.5 wild type incisor. F is a magnified view of the cervical loop from boxed area in E. Green colour represents Sox2, blue identifies DAPI stained nuclei. Dashed line outlines the incisor epithelium. Scale bar represents 100μm.

**
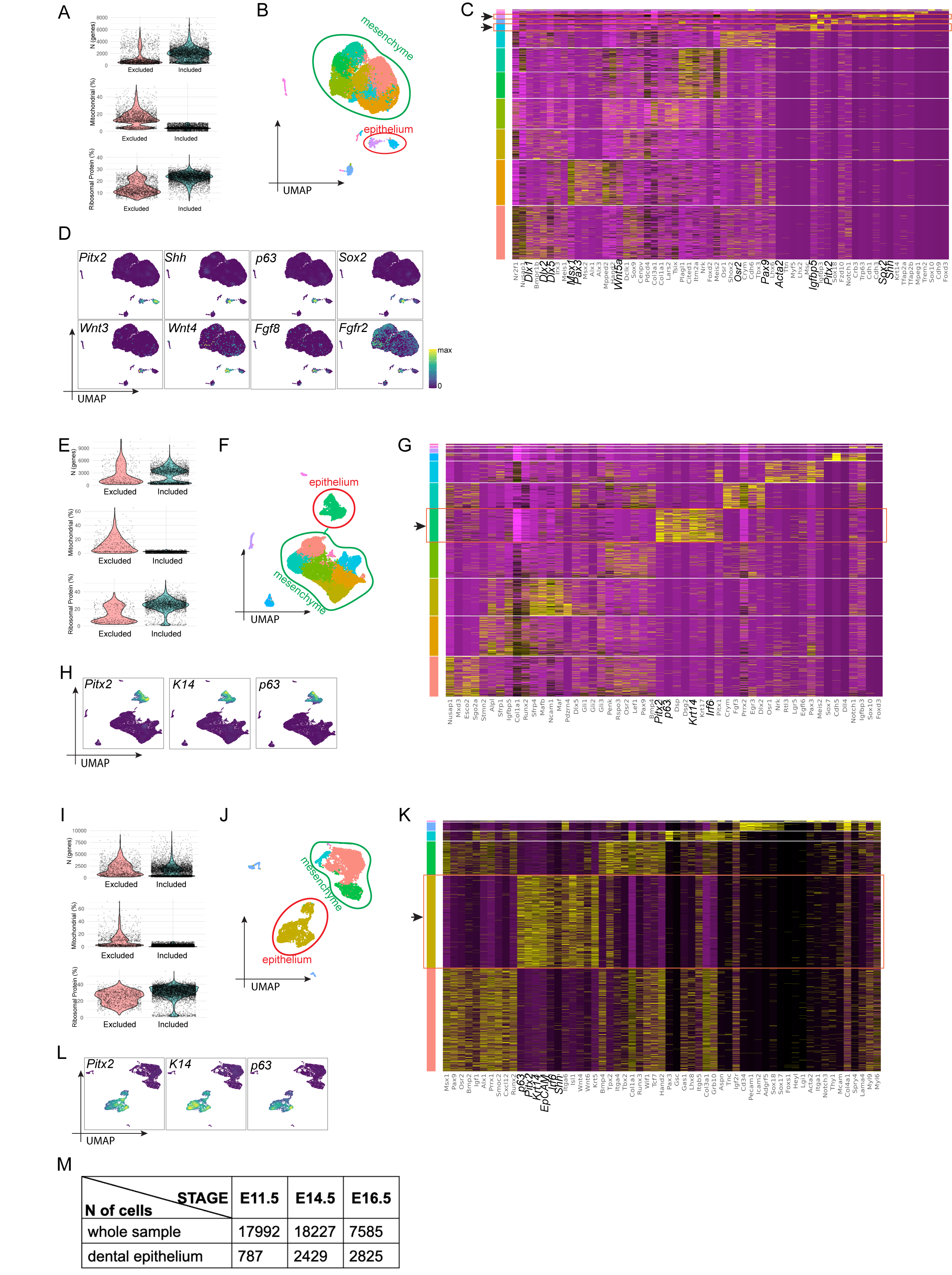
Supplemental Figure 2. Single cell RNA sequencing analysis of the developing incisors.** scRNAseq analysis was performed at the initiation time at E11.5 (A - D), at E14.5 (E - H) and at E16.5 (I - L). Quality controls (A, E and I) and UMAP plot presentation of clusters (B, F and J). Heatmaps showing molecular specificities for each stage (C, G and K) and UMAP plots depicting expression of known markers of dental epithelium (D, H and L). (M) a table showing the total number of cells analyzed in each sample, and the number of cells in each epithelial cluster.

**
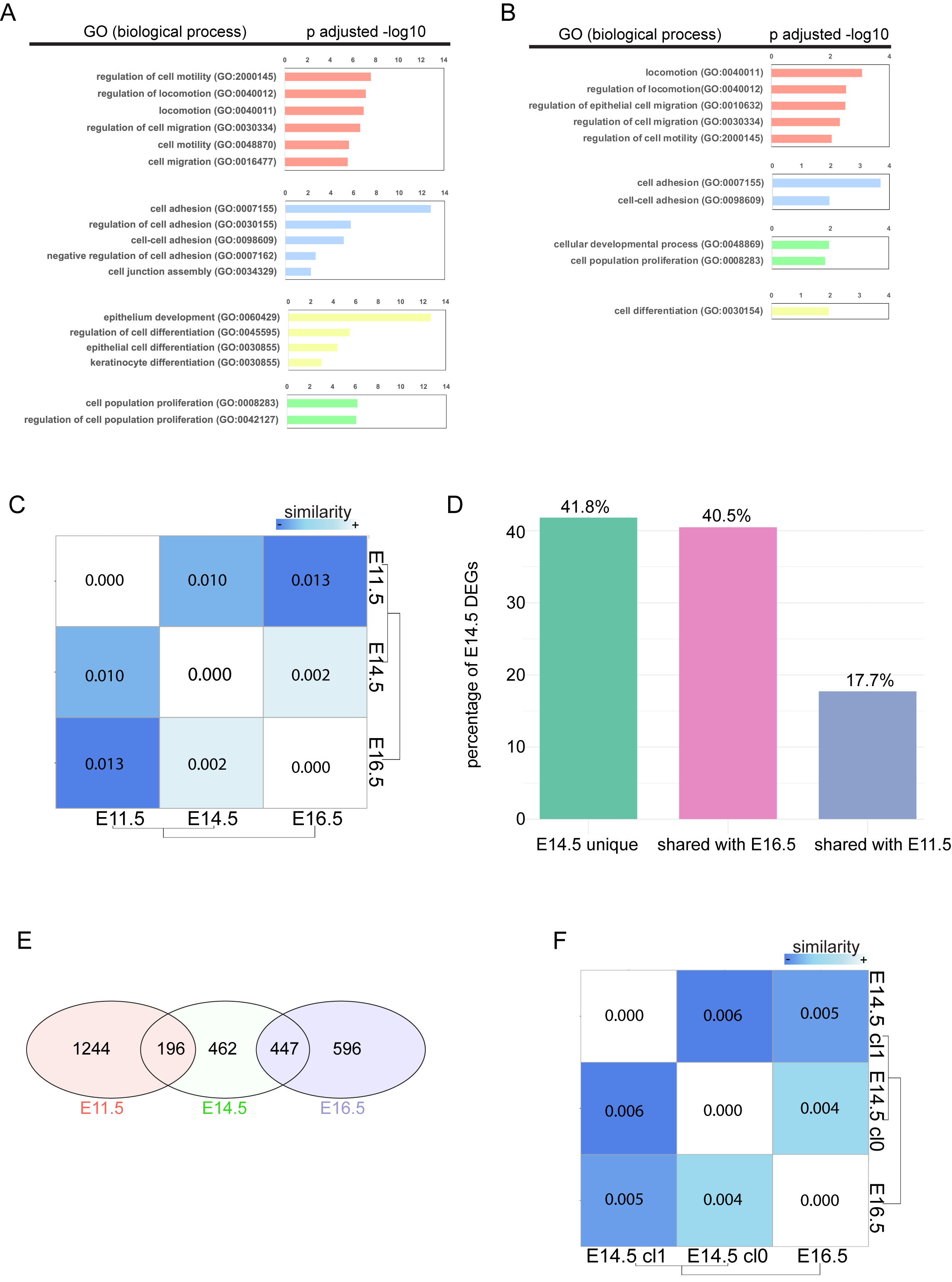
Supplemental Figure 3. Comparative analysis of different stages.** (A, B) Selected gene ontology (GO) biological process differentially found in cluster 1(A) and cluster 2 (B) when compared to cluster 0, and their adjusted p-values. (C) Cell Population distance analysis represented as Mean squared error (MSE) distances between E11.5, E14.5 and E16.5 samples. (D, E) Comparison of the molecular profile of E14.5 stage with the E11.5 and E16.5 stages. Graphic (D) and Venn diagram (E) presentation of shared and exclusive genes between these stages. (F) Cell Population distance analysis represented as Mean squared error (MSE) distances between clusters 0 and 1 from E14.5 and cluster 2 from E16.5.
